# Challenges, Applications, and Recent Advances of Protein-Ligand Docking in Structure-Based Drug Design

**DOI:** 10.3390/molecules190710150

**Published:** 2014-07-11

**Authors:** Sam Z. Grinter, Xiaoqin Zou

**Affiliations:** 1 Informatics Institute, University of Missouri, Columbia, MO 65211, USA; E-Mail: szg4y4@mail.missouri.edu; 2 Dalton Cardiovascular Research Center, University of Missouri, Columbia, MO 65211, USA; 3 Department of Physics & Astronomy, University of Missouri, Columbia, MO 65211, USA; 4 Department of Biochemistry, University of Missouri, Columbia, MO 65211, USA

**Keywords:** protein-ligand interactions, structure-based drug design, molecular docking

## Abstract

The docking methods used in structure-based virtual database screening offer the ability to quickly and cheaply estimate the affinity and binding mode of a ligand for the protein receptor of interest, such as a drug target. These methods can be used to enrich a database of compounds, so that more compounds that are subsequently experimentally tested are found to be pharmaceutically interesting. In addition, like all virtual screening methods used for drug design, structure-based virtual screening can focus on curated libraries of synthesizable compounds, helping to reduce the expense of subsequent experimental verification. In this review, we introduce the protein-ligand docking methods used for structure-based drug design and other biological applications. We discuss the fundamental challenges facing these methods and some of the current methodological topics of interest. We also discuss the main approaches for applying protein-ligand docking methods. We end with a discussion of the challenging aspects of evaluating or benchmarking the accuracy of docking methods for their improvement, and discuss future directions.

## 1. Introduction

In the not-so-distant past, the effects of drugs on disease were known only by empirical observation. A century of subsequent research has revealed many intricacies in the working of cellular receptors and other drug targets, and likewise the methodology of finding small molecules that bind to specific targets has become increasingly complex. This development has been marked by the realization that the interacting surfaces of cellular receptors are chemically active and often flexible, and that these properties tend to be critical to the biological effects of the small molecules, or ligands, that bind to these receptors. Within this climate of complexity, the field of rational drug design emerged to play an important role in the search for new medications [1]. 

An important early step in rational drug design is the identification of a biological target of interest [2]. This target of interest may be as simple as the ligand receptor of an enzyme whose over-activity is associated with disease; a compound that attenuates the enzyme’s action by competitive inhibition would be pharmaceutically interesting. However, there are many other types of drug targets and a variety of chemicals that bind to them (see Table 1 for a list of the most frequently-targeted gene families) [3]. 

**Table 1:** The percent distribution of the gene families targeted by FDA-approved drugs as of 2005. These statistics were compiled by Overington *et al.* from FDA data in 2005 [3].

Portion of Drugs	Family of Drug Target
26.8%	Rhodopsin-like GPCRs
13.0%	Nuclear receptors
7.9%	Ligand-gated ion channels
5.5%	Voltage-gated ion channels
4.1%	Penicillin-binding protein
3.0%	Myeloperoxidase-like
2.7%	Sodium: neurotransmitter symporter family
2.3%	Type II DNA topoisomerase
≈35%	(other)

Rational drug design aims to use knowledge of the biological target of interest to optimize the process of finding new medications. It may be divided into two broad categories: *de novo* drug design, in which a novel compound is designed from scratch, and virtual database screening, in which computational methods are used to search through libraries of small molecules, in order to find those that are predicted to be the most likely to bind to a drug target of interest [1]. *De novo* drug design has the advantage of versatility; only the imagination and the need to synthesize the compound in question limit its conceptual possibilities. However, this advantage can also be a disadvantage. New compounds can prove difficult or expensive to synthesize, constraining the number of new compounds that may be subsequently analyzed by experiment. In addition, predicting the interactions of entirely novel compounds is inherently difficult. The other category, virtual database screening, helps mitigate the synthesis problem by focusing on large databases of synthesizable compounds. 

In virtual database screening, computational techniques are used to search databases of compounds for small molecules predicted to bind to a drug target [4]. Such predictions are not meant to replace experimental affinity determination, but virtual screening methods can complement the experimental methods by producing an enriched subset of a large chemical database; the enriched subset is one in which the proportion of compounds that actually bind to the drug target of interest is increased, compared to the proportion within the whole database [5]. Thus, compounds from the subset that pass the initial virtual screening are found to be pharmaceutically interesting at a higher rate and at a lower cost. 

In principle, the methods used in virtual screening may be applied to any conceivable compounds, but in practice one usually focuses on curated libraries of purchasable or synthesizable compounds, or close analogues of such compounds. Some examples include Accelrys Available Chemicals Directory (Accelrys, Inc., San Diego, CA, USA), eMolecules Database (eMolecules, Inc., La Jolla, CA, USA), and the free ZINC database [6]. 

There are two general types of virtual screening: ligand-based virtual screening and structure-based virtual screening. In ligand-based virtual screening, properties of a set of ligands known to bind to the drug target of interest are used to build a model for the common features believed to be important for a ligand’s biological effects. This model can then be used to find new ligands that share these common features [7]. In structure-based virtual screening, the ligands are modeled as physical entities and scoring functions are used to predict the affinity of the ligand for the binding site of interest [4]. The present review will focus primarily on structure-based methods, but will occasionally refer to ligand-based methods, given the complementary role they often play in the drug design process. 

Structure-based virtual screening typically employs docking software that is designed to explore the possible binding modes of a ligand within a binding site of interest and scoring functions that are used to estimate the affinity of the ligand for the binding site of interest [8,9,10,11]. These sampling and scoring methods will be discussed in more detail in the next section. The scoring of ligands likely to bind to a protein target of interest may also make use of QSAR (Quantitative structure–activity relationship) models, which relate features in the ligand alone or features of the protein-ligand interaction to the biological activities of those ligands [12,13]. Protein-ligand docking methods require a structural representation of the binding site, which may come from X-ray crystal structures, NMR experiments, or homology models [14]. The structure of the small molecules may similarly come from crystal structures, but for large-scale database screening, it is often necessary to model the possible conformations *de novo*. Protein-ligand docking is not usually applied to the whole surface of a protein to predict a ligand’s binding site. Instead, separate methods or biological information about the protein target are used to determine the primary binding site, and the subsequent docking is restricted to this site of interest. Nevertheless there are a few combined methods in which the binding site search and docking procedure are performed simultaneously (also known as blind docking) [15,16]. 

There is a great variety of software packages available for performing protein-ligand docking. Some popular ones include DOCK [17,18,19,20,21,22], AutoDock [23], LUDI [24], FlexX [25,26], GOLD [27], Glide [28,29], and AutoDock Vina [30], in addition to MDock [31,32,33,34,35], developed in our laboratory. An exhaustive review of literature-cited protein-ligand docking software packages was presented in [36]. Depending on factors such as the scoring function and sampling exhaustiveness, the docking software used to perform structure-based virtual database screening may vary greatly in speed, but is often slower than the ligand-based methods. While the ligand-based methods tend to be quite fast, they have the disadvantage that they require a set of ligands known to bind to the target. The ability of ligand-based method to find new active compounds is greatly dependent on the diversity or exhaustiveness of the set of ligands used to build the model [37]. 

In summary, we will review the application, methodologies, and evaluation of the protein-ligand docking approaches. In Section 2, we will introduce the basics of computational protein-ligand docking, discussing the fundamental challenges faced by these methods, and follow with some recent challenges that have been intensely researched in the last two years. In Section 3 we will sample the main approaches used in the biological application of protein-ligand docking methods. In Section 4 we discuss the benchmarks and evaluations used to compare the success of various protein-ligand methods. Finally, we end with some discussion and remarks about the future direction of the field. 

## 2. Challenges in Protein-Ligand Docking

As aforementioned, protein-ligand docking software attempts to sample the possible ways a ligand can be positioned in a protein receptor of interest, and typically provides an estimate of the binding affinity and binding mode of a ligand for the protein receptor [8,9,10]. Docking involves an intrinsic trade-off between the speed of the docking algorithm and its accuracy. In an attempt to achieve higher accuracy, one can employ more advanced scoring functions or more exhaustive sampling of the possible binding modes and flexibility, but these modifications usually add to the computational cost. This tradeoff is very evident in large-scale virtual database screening, in which the number of compounds involved tends to place practical limits on the available computational time per compound. Despite all these challenges, protein-ligand docking methods have enjoyed considerable success in applications [38]. In this section, we will first introduce the basic methodologies of docking in the context of the two fundamental challenges: sampling and scoring. We will then discuss more recent methodological work. 

### 2.1. Scoring Methods

An essential component of docking methods is the scoring function. In protein-ligand docking, the scoring function typically assesses the overall favorability of a protein-ligand complex and is meant to be comparable to the free energy of binding of the protein and ligand [39,40]. There are other attributes than one may want to score for practical reasons such as toxicity and properties related to absorption, distribution, metabolism, and excretion [41]. In addition, even within the sampling algorithm itself it may be advantageous to use more than one scoring function; for example one can use a quick, simple scoring function to discard the worst binding modes before assessing the rest more thoroughly [20,42]. However, accurately predicting binding free energy with a general scoring function, while a very ambitious goal, would revolutionize the utility of the docking methods in drug design and other applications [43]. 

Computing the binding affinities of protein-ligand complexes cannot yet be done very accurately by a general scoring function. The calculation is especially challenging due to the combinatorial explosion of possible conformational states of the flexible protein and ligand, and of the surrounding water molecules and ions. In addition, the binding process involves a balance between many different physical interactions: a flexible ligand may gain favorable interactions upon binding, while simultaneously suffering a substantial entropic penalty as a result of binding. For example, charged polar groups may gain favorable electrostatic interactions when binding, while simultaneously losing favorable interactions with the solvent. Even when polar groups are solvent-exposed, as they like to be, this energetic favorability is partially tempered by the loss of entropic freedom of nearby water molecules. The contribution of each of these interactions can be substantial, yet they tend to cancel each other out; therefore the total binding free energy involves a delicate balance, and inaccuracies in the computation of any one type of interaction can lead to substantial inaccuracies in the computation of total binding free energies [39]. 

Here we discuss the three main types of scoring functions used for docking. Firstly, there are the force-field-based approaches, which attempt to exhaustively model the many types of interactions involved in protein-ligand binding using physics-based functional forms and parameters that are derived from experiments or quantum mechanical simulations. Secondly, there are empirical approaches, in which regression or machine learning methods are used to associate the desired prediction, typically the binding affinity of the complexes, with general features of those complexes such as the number of hydrogen-bonding pairs. Finally, there are statistical potentials, in which energy-like terms are assigned to structural features of protein-ligand interactions based on the frequency with which those features occur in a training set of protein-ligand complexes. 

#### 2.1.1. Force-Field-Based Potentials

Force-field-based potentials can be used in protein-ligand docking as well as molecular dynamics (MD) simulations. They generally include a number of terms representing the various kinds of physical interactions that dominate protein-ligand binding. There are many popular force-field-based potentials in use for various applications, but most of them are quite similar in functional form. The main differences between them are which terms are included in the functional form and which specific values are used for the parameters in those terms. These parameters can be derived for experiment or fitted based on quantum mechanical simulations [44]. Consequently, the entities that are referred to as force fields in docking and MD simulations are typically sets of parameters for use with the functional forms described below. One popular functional form of the force-field based potentials is the one associated with the AMBER molecular dynamics software package [45]. 

The AMBER force fields take the following functional form [46].

*E_AMBER_* = *E_angle_* + *E_bond_* + *E_dihedral_* + *E_non−bonded_* (1)

*E_angle_* and *E_bond_* are harmonic approximations of the bond angle and strain energies, respectively, and *E_dihedral_* is an energy term associated with the dihedral angles of linearly-bonded sets of four atoms (especially, the backbone dihedral angels of proteins). The term *E_non−bonded_* aggregates the non-bonded interactions: a Lennard-Jones 6-12 potential which approximates the van der Waals attraction and Pauli repulsion [47], and an electrostatic potential term. 

The ff94 force field, which uses the AMBER functional form, has been very popular for simulating proteins [44], as have several subsequent versions such as AMBER 99SB force field, which differs from ff94 in the parameters associated with the backbone torsion angles [44]. The general AMBER force field (GAFF) offers parameters suitable for simulating small organic molecules such as drugs [45]. 

The CHARMM force fields are similar the AMBER force fields, but include some additional terms.

*E_CHARMM_* = *E_angle_* + *E_bond_* + *E_dihedral_* + *E_non−bonded_* + *E_improper_* + *E_UB_* (2)

The terms E_bond_, *E_angle_*, *E_dihedral_*, and *E_non−bonded_* have functional forms like those in the AMBER force fields, but the parameter values may differ. The Urey-Bradley term, *E_UB_* [48], is based on the distance between the outer atoms when three atoms are linearly-bonded to each other. The *E_improper_* term provides an energy penalty for improper dihedral angles and helps to control the interconversion of stereocenters. The parameters in *E_UB_* and *E_improper_* can be optimized based on vibrational spectra [48]. The CHARMM22 force field is one of the popular ones that use the functional form defined in Equation (2) and is suitable for modeling proteins [48]. The more-recent CGenFF is suitable as a general force field for small molecules [49]. 

In addition to functional forms and parameterization, the force-field-based approaches are also distinguished by the method of simulating the solvent. The most obvious approach is to model explicitly all of the water molecules in the vicinity of a ligand-receptor and their interactions with the protein and ligand. There are a variety of explicit water models, which are distinguished by the number of sites used to represent the charge distribution of each water molecules: TIP3P uses 3-sites to represent the charge of the oxygen and two hydrogens, TIP4P splits the oxygen into two sites to better represent the charge distribution, and so on [50]. Due to the large number of degrees of freedom of the water molecules, simulating them explicitly is very computationally expensive. 

To simulate some of the effects of the solvent while reducing the computational expense, implicit solvent approximations were introduced. These approximations generally start with the assumption that the solvent can be treated as a continuous dielectric medium with a charge distribution and a resulting electrostatic potential that obeys the Poisson-Boltzmann (PB) equation [51,52,53,54]. The PB equation can be used directly, or alternatively, further simplifications are possible. The most common of these is the generalized Born (GB) model of solvation, in which the protein and ligand atoms are modeled as spheres with a different dielectric constant than the solvent [39,55,56,57,58,59,60,61]. The PB and GB models provide adequate approximations of the electrostatic effects of the solvent. In order to also include an approximation of favorable hydrophobic-hydrophobic interactions, the solvent-accessible (SA) surface area method may be used in combination with the PB and GB models [55]. In this method, the free energy of solvation is assumed to be proportion of the surface area of the solvent accessible atoms, where the contribution of each atom depends on its type. The resulting models, PB/SA and GB/SA respectively, provide high-speed approximations for the major energetic effects of the solvent [62,63,64,65,66,67]. 

To further decrease the complexity, there are empirical solvent methods. In these methods, the electrostatic forces between the protein and ligand are modulated by an empirical distance-dependent parameter that roughly models the tendency of water to screen the electrostatic forces between charged atoms. For one example, this approach was used in DOCK [18]. 

Finally, it is worth noting that the force-field-based potentials mentioned above give estimates of the internal energy of the protein-ligand system in a specific microstate rather than the free energy of binding. In principle, one can use direct integration of the partition function to compute the free energy. Computing the full partition function is typically intractable, but sometimes approximations of the partition function are used [68]. In practice, to estimate the binding free energy using force-field-based potentials, it is necessary to either include the entropic contribution to free energy as an additional approximate term, or to employ umbrella sampling or free energy perturbation methods [69]. 

#### 2.1.2. Empirical Scoring Functions

Given their relatively complex functional forms, the force-field-based approaches described in the previous subsection are computationally intensive. In order to provide a higher-speed alternative, researchers introduced empirical scoring functions [70,71]. Like force-field-based potentials, empirical scoring functions contain terms that are based on structural features and are often inspired by physical interactions. However, empirical scoring functions differ in that the underlying functional form of these terms is simplified in an effort to capture the favorability of an interaction without capturing the underlying physics of the interaction [72]. 

Empirical scoring functions combine features such as hydrophobic contacts, hydrophilic contacts, or number of hydrogen bonds, and parameterize these features as favorable or disfavorable based on regression or machine learning methods. Typically the parameters are optimized to predict the binding affinities of a set of protein-ligand complexes that are used as a training set [70,71]. In this way, empirical scoring functions are reminiscent of the ligand-based models mentioned previously, except that instead of building a specialized model for each drug target, one uses a diverse training set in an attempt to produce a scoring function that can interpolate the binding affinities for drug targets not considered in the training set. Nevertheless, the general performance of empirical scoring functions has been limited by over-simplifications of some of the physical interactions [73]. Some examples of empirical scoring functions include LUDI [24], ChemScore [70], and X-SCORE [74]. 

#### 2.1.3. Statistical Potentials

Besides the empirical scoring functions, there is another type of scoring function that uses a simpler functional form than the ones given in Equations (1) and (2). Statistical potential-based scoring functions (also known as knowledge-based scoring functions) assign energy-like quantities to structural features, based on the frequency with which those features are found to occur in a training set of suitable examples, such as a set of protein-ligand complexes, relative to a reference state [75,76,77,78,79]. Usually, the inverse-Boltzmann equation is used to provide the relationship between the frequency of features and the energy that is assigned to those features. For protein-ligand interactions, the energy assigned to the interaction between ligand atom type *i*, protein atom type *j*, at a distance of *r_k_* (the distance of the k-th bin), can be computed as follows [80].

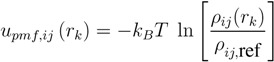
(1)
The quantity *ρ_ij_*(*r_k_*)/*ρ_ij_*_,ref _ is the relative radial density for atom pair type*ij* within the training set and is a function of the binned distance *r_k_*. The density *ρ_ij_*_,ref _ associated with the reference state may be computed using an ideal gas approximation or other approaches [81,82]. 

The derivation of statistical potentials using the inverse-Boltzmann relation is not necessarily physically rigorous, and typically involves some false assumptions. One example is the assumption that the occurrences of features in the training set are conditionally independent of each other, given the energies associated with those features. In applications such as protein interactions and protein structure prediction, the interdependencies neglected by the derivation can manifest in the form of problems such as the excluded volume problem [77]. However, much like naïve Bayes classifiers, statistical potentials based on the inverse-Boltzmann relation have performed well in a variety of applications, regardless of the existence of dependencies within the feature set. Some more recent works have used a multibody approach that reduces this problem [83,84]. 

Another open problem in the implementation of statistical potentials is the reference state problem [77,79,82,85,86]. In order to use the inverse-Boltzmann relation to assign energies to features such as protein-ligand atom pair distances as in Equation (3), it is necessary to define a representative non-interacting state, which provides the frequencies of features one would expect to see in the training set if the features were energetically neutral. A simple ideal gas approximation may be used, and many alternatives have been proposed [81,87,88,89]. Thomas *et al.* introduced an iterative method of deriving a statistical potential that helps avoid the need to define a specific reference state. In this method, which was developed for protein folding, the interaction potentials between residues were iteratively adjusted based on the difference between the residue pair frequencies in the native structures and the residue pair frequencies in a Boltzmann-weighted ensemble of decoy conformations [78]. The extension of this approach to atomic, distance-dependent statistical potentials is non-trivial, due to the involvement of high-dimensional parameter optimization. This challenge was addressed by later methods, which have been applied to protein-ligand interactions, protein-protein interactions, and protein-RNA interactions [31,90,91,92]. 

Finally, there is also the sparse data problem. The inverse-Boltzmann relation in Equation (3) maps the observed frequency of features in a training set to the energies assigned to those features; for features that occur infrequently in the training set, the deriving energies are inaccurate or undefined. Even when very large training sets are available, the problem persists due to physically disallowed states such as very close atom pair distances (*i.e.*, clashes) [93,94]. 

Given the many approaches that have been used to tackle the problems mentioned above and the diverse applications, there are many examples of statistical potentials. Some of the popular ones for protein-ligand interactions include DFIRE [95], DrugScore [96,97], ITScore [31,32], PMF-score [80], and SMoG [98]. 

#### 2.1.4. Summary

The force-field-based potentials, statistical potentials, and empirical scoring functions each offer specific advantages and consequently tend to be used for different applications. Force-field-based potentials separate the types of physical interactions within the system into separate terms, and can therefore provide information on the contribution of these interactions to the internal energy of the system [45,46,48]. Force-field-based potentials can also provide a more detailed simulation of the solvent, especially when explicit water is used. These advantages come at the expense of much greater computational complexity, so force-field-based potentials are not commonly used for high-throughput docking studies. 

Empirical scoring functions [70,71] use a much simpler functional form that permits high-speed implementations for virtual database screening. Empirical scoring functions often give good performance for families of proteins or compounds that are similar to complexes within the training set, but do not tend to generalize well to different protein families. As the number of protein-ligand crystal structures with known affinities increase, the ability of empirical scoring functions to provide good general performance is likely to increase [99]. 

Like empirical scoring functions, statistical potentials use a simple functional form allowing for faster implementations than are typically possible with force-field-based potentials [75,76,77,78,79]. Unlike empirical scoring functions, the interaction terms in statistical potentials are not typically fitted to reproduce the affinities associated with a set of protein-ligand complexes; instead, the terms are derived based on a presumed relationship between the frequency of features in a training set and the energies associated with those features. The derivation of a statistical potentials therefore is less prone to over-fitting, and the performance can generalize well to protein-ligand complexes that differ from those in the training set. Statistical potentials are advantageous when low computational complexity is desired and when the performance of the potential is expected to generalize well to cases for which the training set provides poor coverage. 

There have been some efforts to combine different scoring functions in a way that provides a compelling combination of the advantages in each scoring function. An early example of a consensus scoring function may be found in [100]. Other examples of the consensus scoring approach include MultiScore [101], X-Score [74], and VoteDock [102]. The scoring function presented in [94] to deal with the sparse data problem can also be considered a consensus approach. 

### 2.2. Sampling Methods

The other fundamental challenge facing protein-ligand docking methods is sampling. Protein-ligand binding involves changes in the relative orientation and conformation of the ligand, as well as possible conformational changes to the protein. Docking software attempts to sample these possible changes with varying degrees of exhaustiveness [103]. 

The simplest approach for sampling the possible ligand binding modes is rigid docking; the docking software can simply explore the six degrees of translational and rotational freedom and filter those with poor shape complementarity before final scoring. This approach was used by older versions of DOCK [104] as well as MDock [31,32]. Ligand flexibility can still be considered by such software by pre-computing ensembles of putative ligand conformations, using software such as OMEGA (OpenEye Scientific Software, Santa Fe, NM, USA) [105,106], and rigidly docking each conformation to the protein receptor of interest. 

There are also docking approaches that sample the possible ligand conformations on-the-fly. One method of on-the-fly sampling is the incremental construction method, also known as the anchor-and-grow method. A rigid central portion of the ligand is placed in the binding site, and the rest of the ligand is incrementally grown from this rigid anchor, filtering out those possibilities that clash with the protein receptor during the process [20,107]. DOCK uses this approach [20]. Similar to above, there are also fragmentation methods, in which multiple rigid fragments are placed within the binding site and the docking software attempts to link these pieces together to reconstruct plausible conformations of the target ligand. LUDI uses this approach [24]. 

Another approach for sampling ligand conformations is the hierarchical docking method. In this approach, low-energy conformations for each ligand are pre-computed and aligned so that as many atoms as possible are identically-positioned. Each ensemble of pre-generated ligand conformations is organized into a hierarchy so that similar conformations are similarly positioned within the hierarchy. Then, for each possible translation and rotation of the ligand, the docking software makes use of the hierarchical data structure to simultaneously prune or filter sets of conformations that are not sterically possible for the given translation and rotation. For example, if an atom near the rigid center of the ligand is found to clash with the protein in a given rotation/translation, the method can confidently reject all of the descendent conformations in the hierarchy for that rotation/translation, because the descendants must contain the same clash, without having to sample each descendant conformation individually [108]. The Glide software package uses hierarchical filters during ligand sampling [28,29]. 

In addition to these methods of sampling ligand conformations, there are methods to handle protein flexibility. One simple approach is to rigidly dock the ligands to several putative conformations of the protein, to represent some of the protein’s conformational variability [33,34,109,110]. Another approach, which may be used alone or in conjunction with ensemble docking is energy minimization. Minimization may be performed using Monte Carlo methods or gradient descent minimization to help simulate some of the induced fit that occurs when a ligand binds to a protein receptor [111]. Finally, one can also attempt to explore the conformational space of critical residues of the protein, using methods analogous to the ligand methods mentioned before. For example, AutoDock4 [112] and AutoDock Vina [30] can adjust the rotatable bonds of critical residues in order to simulate protein conformation changes during binding. 

### 2.3. Recent Topics

While sampling and scoring constitute the fundamental challenges in docking, much research focuses on more specialized topics. We do not attempt to exhaustively review all of the active topics, but instead sample a few topics that have received a large amount of recent attention. 

#### 2.3.1. Structural Water

As mentioned in Section 2.1.1, water plays an important role in protein-ligand binding, often counteracting the attractive interactions between the protein and ligand and resulting in a delicate balance of forces that is a difficult to model accurately [113]. One aspect of the solvent that has been increasingly recognized as a major actor in protein-ligand interactions is structural water. In the vicinity of the protein and ligand, molecules of water may become bound or semi-bound in certain favorable positions, stabilized by hydrogen bonds, and these structural water molecules can play a critical role in the stability of a protein-ligand interaction [114,115]. In work by Lie *et al.*, modeling structural water molecules was found to increase the accuracy of docking simulations, up to a binding mode success rate of 67% [116]. Other work involving the inclusion of structural or bridging water molecules into high-accuracy protein-ligand docking simulations also shows improvements in accuracy [117]. In one paper, the ability of Rosetta) [118] to reproduce the binding mode of the HIV-1 protease/protease inhibitor crystal structures was investigated. It was found that the inclusion of just a single structural water molecule in the interface was crucial for accurate prediction of an inhibitor binding pose [119]. 

Docking methods improved by structural water simulation have seen practical applications, such as an inverse docking application in [120]. 

#### 2.3.2. Ligand Promiscuity

It has been well-recognized that drugs may bind to many targets with significant affinities, and that this drug promiscuity gives rise to a complex polypharmacology with clinical relevance to the toxicity and side effects of pharmaceuticals [3,121]. The utility of considering such ligand promiscuity early in the drug design process has already been well-recognized. A review by Taboureau *et al.* noted the regulatory recommendation that all new drug candidates be tested for their potential to block human Ether-a-go-go Related-Gene (hERG) potassium channel, given the substantial risk of cardiotoxic side effects such as arrhythmias [122]. They considered *in silico* screening to be a useful step in identifying cardiotoxic leads before they are given larger investments. 

Besides the prediction of toxicity and side effects, the tendency of ligands to bind to several sites presents another challenge: if a ligand binds tightly somewhere, even near the desired binding site, it may still not substantially affect the drug target in question. Gowthaman *et al.* point out that this is particularly important for non-traditional drug targets, such as a target within the interface of protein-protein interactions. For such targets, compounds that bind are often inadequate, if they do not bind in a sufficiently buried manner to achieve good ligand efficiency [123]. Perez-Nueno *et al.* introduced a ligand-based approach that uses shape matching to identify promiscuous ligands [124]. 

One the other hand, particularly for multifaceted diseases such as cancers or metabolic disorders, it may be desirable for a drug to bind to multiple targets. Peng *et al.* review the chemogenomics approaches in which a spectrum of a ligand’s interactions with many drug targets is predicted by structure-or ligand-based methods. Using these approaches, one can attempt to increase those interactions within the spectrum that are desired while simultaneously reducing unwanted interactions [125]. 

#### 2.3.3. Accurate Models of the Protein Receptor

Docking studies often employ comparative (or homology) models of the protein target that are based on the crystal structures of homologous proteins. The methodology behind building homology models is outside the scope of this review, but here we remark on the popularity of the approach in practice. It is notable that a number of recent successful virtual screening projects used homology models of the protein receptor, and for some the best template for homology modeling was fairly divergent from the target structure, in terms of percent sequence identity [126,127,128,129,130,131,132]. 

Nguyen *et al.* investigated the accuracy of predicting ligand binding modes in comparative models of G-protein coupled receptors. The researchers found that for the best models with template structures over 50% sequence identity, the accuracy of binding mode prediction was within 2.9 Å RMSD (root-mean-squared standard deviation) from the native experimental structure on average. In cases of low sequence similarity, it is challenging to produce a homology model with sufficient accuracy to use as a basis for virtual screening [133,134], but percent sequence identity is not the only useful metric. It has also been suggested that choosing a template based on ligand occupancy can yield a better homology model for docking than choosing one based on percent sequence identity [135]. 

## 3. Protein-Ligand Docking Approaches

Having introduced structure-based drug design and the methodologies of protein-ligand docking, we will now sample the most common research approaches in which such methods have been applied. 

### 3.1. Screening for New Inhibitors

Docking methods have a long and successful history of identifying new protein inhibitors and enriching compound databases in structure-based virtual screening. Here we discuss some examples of this common application. 

Recently, Mahasenan *et al.* used structure-based virtual screening to identify new inhibitors of maternal embryonic leucine zipper kinase (MELK), an important kinase target known to be involved in several types of cancer. As signaling molecules, kinase targets tend to be challenging for docking methods due to their tendency to undergo major conformation changes induced by ligand binding [136]. Their three discovered inhibitors vary in affinity from 0.37 µM to 18 µM, and may have future applications in diseases involving mis-regulation of MELK [136]. 

In another recent work, Heusser *et al.* performed a virtual screening study of *Gloeobacter violaceus* ligand-gated ion channel (GLIC), a bacterial homolog of GABA*_A_* receptors, to search for compounds that bind to the same site as the anesthetic propofol. Among a database of commercially available compounds, 29 compounds were experimentally tested of which 16 were found to exhibit significant inhibition of GLIC relative to dimethyl sulfoxide. The active compounds were further tested on GABA*_A_* receptors. One of the compounds, like propofol, was found to inhibit both GLIC and GABA*_A_* receptors, suggesting that the GLIC receptor may be a plausible model system for GABA*_A_* receptor ligands. 

In a third example, Tahir *et al.* used MODELLER [137] to build a homology model of TNFRSF10B protein, which is believed to inhibit tumor formation [130]. In an effort to further understand this protein, they also used protein-ligand docking to screen compounds from the Mcule compound database [138] for new potential inhibitors [130]. 

In a final example, a series of substituted heteroaromatic piperazine and piperidine derivatives were found through virtual screening based on the structure of human enterovirus 71 capsid protein VP1. The preliminary biological evaluation revealed that two of the compounds (8e and 9e) have potent activity against EV71 and Coxsackievirus A16 with low cytotoxicity [139]. 

### 3.2. Hybrid Approaches for Drug Design

The structure-and ligand-based methods of performing virtual database screening are not simply competing alternatives to perform the same task. They each have unique strengths and weaknesses and can therefore play a complementary role in the drug design process and other applications. Such hybrid approaches have become increasingly popular [140]. Here are a few recent examples. 

One example of a hybrid approach may be found in Ahmed* et al.* In this work, the binding profiles of the spherical C 60 version of fullerene and its derivatives were investigated. Aside from the remarkable physicochemical characteristics of these molecules, fullerene and its derivatives are increasingly investigated for their unique biological effects [141]. The hybrid approach used by Ahmed *et al.* included quantum-mechanical calculations, protein-ligand docking and QSAR. They used quantum-mechanical calculations to determine geometries, dipole moments, orbital energies, and other parameters of the fullerene derivatives. They used protein-ligand docking software including AutoDock Vina [30] and Schrödinger Glide [28,29] to search for possible binding modes of the fullerene derivatives’ interactions with HIV-1 protease and to identify which residues of HIV-1 protease tend to be involved in the binding. They also compared the docking scores with experimental binding affinities. Finally they used genetic algorithms to choose a suitable QSAR model predictive of the fullerene derivatives’ binding activity. The most important features in the QSAR model were found to be the 3D-molecular geometry of the fullerene derivative, its number of ring systems, and its specific topology [141]. 

Another work that used a hybrid approach of structure-and ligand-based methods can be found in a study identifying four inhibitors of heat shock protein 90 (Hsp90), which is an important chaperone protein and anticancer drug target [142]. In this work, the researchers built a QSAR model to perform ligand-based virtual screening [142], and used a combined ligand-based/structure-based protocol to screen 1785 compounds for their predicted ability to bind to Hsp90 [143]. 80 of the predicted compounds were further evaluated by experiment and found to inhibit Hsp90 with IC50 values between 18 and 63 µM. The compounds contain possible new molecular scaffolds capable of inhibiting Hsp90 [142,143]. 

The last example of the hybrid approach that we will mention here involved DNA G-quadruplex structures, which are found in some critical positions within the genome such as near the telomeres and gene promoter regions. Unsurprisingly, they are involved in cellular aging and cancers [144]. Alcaro *et al.* used a hybrid approach to screen a database of commercially available compounds for their predicted ability to bind G-quadruplex structures. Before this work, there were already a variety of knowns binders for G-quadruplex structures. They first screened over one million compounds from the ZINC database [6] using ligand-based methods that compared the compounds in this database to the known binders using both 2D-similarity and 3D-similarity methods. The compounds which passed this first screening were then investigated using ensemble docking simulations on a few of the conformations of telomeric G-quadruplex structures that have been structurally characterized. They analyzed the compounds with the highest docking consensus score using several experimental techniques, and determined that they had found a new G-quadruplex binding moiety [144]. 

### 3.3. Mechanistic Studies Using Inverse Docking

Virtual database screening studies do not always start with the identification of a drug target of interest. Often, one is interested in a compound that is known to have an important biological effect, but for which the underlying molecular mechanism is unknown [145]. Consequently, rather than looking for small molecules that bind to a binding site of interest, protein-ligand docking methods may instead be used to perform the inverse search, called inverse docking [146,147]. Inverse docking involves some additional challenges. Relative scoring of protein-ligand complexes that differ according to the protein rather than the ligand is challenging for a number of reasons. Firstly, one needs structures or models of the protein receptors to be screened, but the structures of many proteins have not been solved. This necessitates the laborious process of gathering those proteins relevant to the research in question and determining the location of the binding sites. Alternatively, one may use a curated repository of known drug targets, such as the Potential Drug Target Database [148]. Secondly, proteins are often found to exist in several closely related isoforms, so the scoring function in inverse docking is challenged by the need to rank these subtle differences [149]. Thirdly, scoring functions are usually validated on benchmarks that determine their ability to accurately rank entirely different protein-ligand complexes, or many ligands against a smaller number of proteins. Benchmarks do not usually contain many examples of the same ligand docked to many different proteins, so the performance of most docking methods is more doubtful in this application. Despite these challenges, inverse docking is a popular and useful approach. 

A recent application of inverse docking may be found in [150]. Some plant-derived isoprenoids have antiparasitic effects but the relevant molecular targets of these compounds were unknown. Noting the mortality of leishmaniasis, especially in some tropical regions due to the poor availability of resources to fight drug-resistant parasites, Ogungbe and Setzer used an inverse docking approach to investigate the underlying molecular mechanism of the relevant antiparasitic isoprenoids. Specifically, they compiled the known protein targets of the drugs used to treat Leishmania and docked the isoprenoids of interest to these proteins in order to predict which of the isoprenoids may share similar targets and to offer some clues regarding their functional mechanisms [150]. 

## 4. Docking Benchmarks and Evaluation

Benchmarking plays an important role in the development and improvement of docking methodologies [40]. Public databases combining crystal structures of protein-ligand complexes with experimentally-determined affinity data provide a standard way of assessing the accuracy of the binding mode predictions and binding affinity predictions of protein-ligand docking methods [72,151,152]. In addition to the standard benchmarks, there are prospective evaluations for protein-ligand interaction predictions, also called blind competitions. These prospective evaluations play an important role in the improvement of docking methods by validating new methods on targets that were unknown to the researchers at the time the methodology was developed [153,154,155]. Here we discuss a number of the challenges in benchmarking docking methods. 

### 4.1. Making Testable Predictions

It would be ideal for docking scoring functions, sampling schemes, and other methodologies to be tested in prospective studies in which the targets of the benchmark were unknown at the time the methodology was developed. Such prospective evaluations are not always available when new methods are introduced. In such cases, a rigorous experimental design can help ensure trustable evaluations, especially with regard to the independence of the benchmark from the development of the methods. Examples of prospective evaluations of protein-ligand docking methods include CSAR, the Community Structure Activity Resource [153,155], and OpenEye SAMPL [156]. 

### 4.2. Assuming Lack of Knowledge of the Native, Bound Conformation

In practice, docking methods are unable to rely on the availability of structurally accurate knowledge of the bound, native conformation of a protein binding site and ligand, due to the conformational changes that occur during binding. A realistic evaluation of docking would require the method to dock an arbitrary conformation of the ligand to either a ligand-free crystal structure of the protein, or if none are available, then a crystal structure bound to a different ligand than the one being docked. This allows the docking software to be tested for the ability to either simulate the induced fit of binding, or test the success of a smoother scoring function designed for soft docking. Examples of recent evaluations of docking methods that included unbound evaluations may be found in [157,158]. 

In addition to the change in protein conformation associated with the induced fit of protein-ligand binding, docking methods also have to deal with the flexibility of ligands. To evaluate scoring function performance in flexible binding mode predictions, one approach is to pre-generate many decoy binding modes for a ligand in the vicinity of the binding site, and test the ability of the scoring function to distinguish between the native pose and decoys. This approach was used for decoy sets that extend the CSAR benchmark [152]. 

Korb *et al.* suggested that this approach of testing docking scoring functions with predefined sets of decoy ligands is not adequate for distinguishing a scoring function that performs well in practice from one that performs poorly. The reason is simple: in docking, sampling that is sufficient to identify the native pose and conformation must be very thorough, and so in practice many more diverse poses and conformations are considered during docking than are typically generated for the decoy poses generated for scoring function evaluation [159]. It seems that this problem could be mostly avoided by ensuring that the generated decoys are numerous and diverse, or entirely avoided by testing scoring functions simultaneously with sampling, as in realistic practice [160]. 

### 4.3. Assessing Binding Mode Predictions Involving Symmetric Molecules

Another challenge in evaluating docking methods is the need for special handling of symmetric molecules when evaluating binding mode predictions. The binding mode predictions of a docking method are commonly evaluated using the root-mean-squared standard deviation (RMSD) of atom positions between the known native binding mode of a ligand and its predicted mode according to the docking method. However, comparing the atom positions between two structures of a ligand requires mapping the atoms in the native ligand conformation to the atoms in the docked conformation of the ligand. Due to the symmetry of entire molecules, or substructures within them, these mappings can be ambiguous and a naïve treatment of binding mode evaluations can consider a perfect binding mode prediction to be a poor prediction. Recently, work by Allen *et al.* addresses this problem by using the Hungarian algorithm. The Hungarian algorithm can be used to find the optimal mapping of two graphs under a cost function, and in addition to other applications in chemical informatics, has recently been used by Allen *et al.* to find the optimal mapping between two molecules in RMSD calculations, ensuring that a correct binding mode prediction will be recognized as such [161]. This method has been implemented within DOCK 6 [22] and we anticipate its wide adoption. 

## 5. Conclusions

The methods used to simulate the binding of proteins and small molecules face substantial challenges. Two of the fundamental challenges are sampling and scoring [8]. Protein-ligand interactions involve a delicate balance of competing forces, and these forces may occur between flexible structures that can reposition themselves in far too many combinations to sample exhaustively. Another challenge is the need to account for structural water in approximative models of the solvent [114]. In addition, there is demand for methodologies that can adequately evaluate the wide spectrum of possible binding partners of a given ligand; this is especially important for designing drugs that maximize efficacy while minimizing side effects and toxicity [3,121]. Finally, there is also a need for rigorous, wide evaluations of new docking methodologies, which are rapidly being introduced [40,153]. 

Another important component of protein-ligand binding that is sometimes neglected is the effect of entropy. Rigorous computation of the entropic contribution to binding free energy is intractable for large molecular systems such as protein-ligand complexes. Some approximations have been introduced to deal with entropy [35,162,163], but many of the most popular docking scoring functions for structure-based virtual screening either ignore this important component of protein-ligand binding free energies or use overly simplistic empirical approximations. Future efforts to find computationally efficient ways to include the effect of entropy are likely to play a crucial role in the future advancement of docking methodologies. 

Despite all the challenges, protein-ligand docking has a long and successful history of practical applications including newly discovered enzyme inhibitors, receptor antagonists and agonists, ion channel blockers, as well as the subsequent approval of new drugs discovered with the help of structure-based drug design. Docking methods have provided new mechanistic insights into protein-ligand binding mechanisms, and have also helped investigate the influence of protein mutations on ligand binding, offering clues regarding the mutations that enable the robust survival of drug-resistant pathogens. As the continued increases in computational power expand the practical applications of molecular models, the field is quickly advancing, with approximations that offer a better tradeoff between accuracy and computational cost. These efforts will undoubtably lead to many more intriguing applications into the future. 
